# Working conditions and mental health functioning among young public sector employees

**DOI:** 10.1177/14034948211045458

**Published:** 2021-10-05

**Authors:** Jaakko Harkko, Hertta Ranta, Tea Lallukka, Hilla Nordquist, Minna Mänty, Anne Kouvonen

**Affiliations:** 1 Faculty of Social Sciences, University of Helsinki, Finland; 2 Department of Public Health, University of Helsinki, Finland; 3 South-Eastern Finland University of Applied Sciences, Finland; 4 Unit of strategy and research, City of Vantaa, Vantaa, Finland; 5 Centre for Public Health, Queen’s University Belfast, UK

**Keywords:** Mental health functioning, mental disorders, job demand, job control, physical workload, working conditions, municipal employees, younger employees, SF-36, mental component summary

## Abstract

*Background:* The associations between adverse working conditions and mental disorders are well established. However, associations between adverse working conditions and poor mental health functioning is a less explored area. This study examines these associations among younger public sector employees of the City of Helsinki, Finland. *Methods:* We use data from the Young Helsinki Health Study with a representative sample of the employees of the City of Helsinki, aged 19–39 years (*n*=4 217). Mental health functioning was measured with mental composite summary of the Short Form 36. Working conditions included factors related to both the psychosocial (job control and job demands) and the physical work environment (physical workload). To examine the associations, we used logistic regression models with adjustments for socio-demographics, other working conditions and health-related covariates. *Results:* After adjustment for sociodemographic characteristics, poor health, health behaviours and other occupational exposures, high job demands (OR=1.69; 95% CI=1.45–1.97) and low job control (OR=1.65; 95% CI=1.40–1.94) were associated with poor mental health functioning. High physical workload was not associated with the outcome (OR=0.87; 95% CI=0.72–1.05) after the adjustments. ***Conclusions:* Adverse psychosocial working conditions were associated with mental health functioning, whereas physical working conditions were not. As impaired functioning is likely to cause health-related lost productivity and can lead to work disability, further research and interventions with a balanced approach focusing on both psychosocial working conditions and mental health functioning are recommended.**

## Background

Mental disorders impose a heavy toll on employees, employers and society in general through impaired functioning in the workplace, health-related lost productivity and increased costs of work disability [1, 2]. While employment generally supports mental health [3, 4], adverse psychosocial working conditions, such as high job demands and low job control, have been associated with common mental disorders [5–7] and sickness absence [8, 9] among all age groups of the working population. Psychosocial risk factors have the highest explanatory role in the relationship between adverse working conditions and mental disorders, but the contribution of adverse physical working conditions, such as physical workload, on mental disorders has also been shown in previous studies [10, 11]. While previous studies have found working conditions to be associated with mental health or mental disorders, there is relatively little research on whether working conditions could affect health-related functioning.

Mental health functioning refers to how mental health allows or restricts everyday activities and social participation [12, 13]. In the context of working life, functioning is a useful conceptualisation of health because individuals’ activities and participation in social environments may be more strongly associated with work performance, disability benefits and return to work than psychological symptoms or mental disorders individually [12, 14]. Despite the potential practical benefits of using mental health functioning in policies affecting working conditions or increasing the accuracy of sickness absence predictions, there is very little research on working conditions and mental health functioning, i.e. mental health-related wellbeing and individuals' social roles in the workplace.

From the perspective of health promotion and prevention of mental disorders, still another apparent deficiency in the current knowledge base is that the results of the studies are derived mostly from data collected from middle-aged or age-non-discriminant populations [7, 15]. There are several reasons to focus on the mental health functioning of the younger working populations. Common mental disorders are the most common health problems experienced by young adults in the OECD countries [16], and three-quarters of all mental disorders are established by early adulthood [2]. Besides etiological considerations, prior studies have found differences between the younger and midlife and older employees in how the demand—control model applies to them [17], in mental health symptoms [18], as well as in mental health-related sickness absence [19, 20]. Therefore, it is essential to widen the knowledge base of the relationships between working conditions and mental health functioning, particularly among different age groups. This study focuses on younger employees aged below 40 years. Mental health functioning may provide valuable information for the assessment of psychosocial and physical work environments from the perspective of disease prevention and the prevention of work disability in this age group.

## Aims

To our knowledge, prior studies have not examined whether psychosocial and physical working conditions are associated with mental health functioning among younger employees. We hypothesise that adverse working conditions are associated with poor mental health functioning. We further hypothesise that the effects of different working conditions may be interrelated. In addition, we hypothesise that the associations between working conditions and mental health functioning are affected by poor health and adverse health behaviours. In the present study we focus on the association between working conditions and poor mental health functioning, i.e. potential differences between people with varying working conditions in their risk for poorer functioning. Focusing on poor mental health functioning may also reveal clearer differences than when mental health functioning is used as a scale as an earlier study has suggested that health functioning may have non-linear associations with other mental health measures so that the associations are stronger at the lower end of mental health functioning continuum [13].

## Methods

### Study population

This study is a part of the Young Helsinki Health Study, which is a cohort study investigating social and work-related determinants of health and wellbeing among younger municipal employees [21]. The survey is a new extension to the established Helsinki Health Study (HHS), a cohort study following midlife and older employees of the City of Helsinki since 2000 [22]. The City of Helsinki is the largest employer in Finland with c. 37,000 employees and an occupational structure which covers both manual and non-manual jobs and hundreds of occupational titles. The largest employment sectors are health and social care and education, resulting in a female-dominant employee population.

Survey data was collected in 2017. The target population of the survey was all employees of the City of Helsinki, Finland, who were aged 18 to 39 years [21]. The overall response rate was 51.5% (*n* = 5898). After omitting respondents with incomplete data for any of the study variables (*n* = 1690/28.6%), the final analytic sample consisted of 4217 participants. Included participants had a higher prevalence for poor mental functioning compared with excluded participants, whereas adverse working conditions were more common in excluded participants compared with those who were included in the current analyses (data not shown).

The protocol for the Helsinki Health Study was approved by the Ethics Committees of the Department of Public Health of the University of Helsinki and the authorities of City of Helsinki. The research was consistent with the values expressed in the Declaration of Helsinki.

### Outcome

We measured mental health functioning with the mental component summary (MCS) of the 36-item Short Form Health Survey (SF-36). The SF-36 is a standardised and well-validated health metric for examining the overall health and burden of ill-health of both general and patient populations. The MCS scale integrates the vitality, role limitations caused by emotional problems, general mental health and social functioning subscales of SF-36 [13]. The MCS scale ranges from zero to 100, where the higher scores indicate better mental health functioning [13]. In this study, we dichotomised the score to indicate poor mental health functioning using the lowest quartile in MCS as a cut-off point [23, 24], resulting in cut-off points of 41.9 for women and 43.4 for men. The values below the mentioned thresholds were defined as poor mental health functioning. In the general US population, in the age group of 25–34 years, the corresponding values were 41.9 for women and 45.1 for men [13].

### Predictors

We used three measures for working conditions. To measure job demands and job control, we used the Framingham version of Karasek’s Job Content Questionnaire [25]. The measure of job control consisted of nine items on decision authority and skill discretion. The measure of job demands consisted of five items on excessive work, conflicting demands, insufficient time to work, fast pace and working hard. Job demand and job control included items with five response alternatives from fully agree to fully disagree. For each instrument, we calculated the mean of the individual items. Missing values were replaced by item modes for those having responded to at least four job demands questions and eight job control questions. Cronbach's Alphas, i.e. scale reliability coefficients, were calculated and were 0.74 for job demands and 0.78 for job control. We followed the most commonly used operationalisation and used median values as cut-off points [7]. For job demands, values below the score 3.50 were classified as ‘low’ and those above as ‘high’ (i.e. adverse). For job control values above the score 3.67 were classified as ‘high’ and those below as ‘low’ (i.e. adverse), respectively.

To measure physical working conditions, we used an instrument developed by the Finnish Institute of Occupational Health [26]. The measure of physical workload consisted of four items from this instrument found to measure the physical workload of the employees among the midlife and older employees of the City of Helsinki [10]. The items included were awkward working positions, rotation of the back, repetitive movements and heavy physical effort or lifting and carrying heavy objects. The items had four response alternatives from fully agree to fully disagree. We calculated the mean of these items for those with at least three item responses. Cronbach's Alpha for this measure was 0.86. We used operationalisation that has been used in many previous studies and dichotomised physical workload at the highest quartile [10, 27]; the values above the score 3.00 were classified as ‘high’ (i.e. adverse) and those below as ‘low’.

### Covariates

We controlled for possible confounding caused by sociodemographic factors, poor health and adverse health behaviours. Sociodemographic covariates used in the analysis were gender, age (<30/⩾30), marital status (married or cohabiting/other), having children (yes/no) and occupational class (managers and professionals/semi-professionals/routine non-manual workers or manual workers). For occupational class, we used a classification consistently used in HHS.

Obesity was operationalised by body mass index (BMI) 30 or higher. Insomnia symptoms (yes/no) were defined as having experienced at least one out of four insomnia symptoms from the Jenkins Sleep Questionnaire (JSEQ) [28] in at least half of the nights during the previous month. Adverse health behaviour was operationalised as binge drinking (six or more units of alcohol on a single occasion at least once a month/others) and smoking (daily smoking/others).

### Statistical methods

We used logistic regression models to analyse the associations between working conditions and poor mental health functioning. We present odds ratios (OR) and their 95% confidence intervals (CI). The results are presented for the studied working conditions (low job control, high job demands and high physical workload) having the group with the lowest risk for the outcome as the reference category. We sequentially modelled the data adjusting for different clusters of covariates. The first model, presenting estimates for each working condition individually, was adjusted for gender, age, marital status, having children and occupational class. In the second model, we added two other adverse working conditions to the first model. In the third model, the indicators of poor health and adverse health behaviours were added to the first model. The fourth, the full model, consisted of all the study variables. Women and men were pooled together as we found no statistically significant gender-exposure interaction effects.

We ran two sets of sensitivity analyses. First, we replicated the analyses by including participants with missing values on the covariates. Second, we re-ran the analyses using predictors divided into quartiles. The results from the sensitivity analyses and the Pearson’s correlation matrix for the outcome and predictors are presented in Appendices 1–3.

All analyses were carried out with the Stata 16 software package.

## Results

### Descriptive results

Table I shows descriptive statistics and the distribution of poor mental health functioning among women and men by working conditions, sociodemographic characteristics, poor health and adverse health behaviours. Among the 4208 participants (78% women), the age ranged from 19 to 39, with a mean of 32 years. Among women, those aged 30–39 years had a higher prevalence for poor mental health functioning. Among men, poor mental health functioning was more prevalent among those under the age of 30. In both women and men, those who were married or cohabiting reported less poor mental health functioning. There was no difference in the prevalence of poor mental health functioning between occupational classes. Those with poor health and adverse health behaviours had a higher propensity for reporting poor mental health functioning.

**Table I.** Descriptive statistics and the distribution of poor mental health functioning (MCS, Q1*) among men and women by working conditions, sociodemographic characteristics, poor health and adverse health behaviours.

	Women			Men		
	*N*	%	MCS Q1, %	*N*	%	MCS Q1, %
All	3274	100.0	25.0	943	100.0	25.0
*Exposures*						
Job demands						
Low	1650	50.4	20.1	655	69.5	21.2
High	1624	49.6	30.0	288	30.5	33.7
Job control						
High	1553	47.4	20.6	467	49.5	19.7
Low	1721	52.6	29.0	476	50.5	30.3
Physical workload						
Low	2458	75.1	24.1	788	83.6	25.3
High	816	24.9	27.8	155	16.4	23.9
*Covariates*						
Age						
19–29	1102	33.7	29.2	254	26.9	22.4
30–39	2172	66.3	22.9	689	73.1	26.0
Occupational class						
Managers or professionals	905	27.6	24.1	283	30.0	25.1
Semi-professionals	1376	42.0	24.9	291	30.9	25.8
Routine non-manual workers and manual workers	993	30.3	26.0	369	39.1	24.4
Marital status						
Others	1266	38.7	31.0	309	32.8	33.0
Married or cohabiting	2008	61.3	21.3	634	67.2	21.1
Has children						
No	2052	62.7	27.8	596	63.2	27.5
Yes	1222	37.3	20.4	347	36.8	20.7
BMI >30						
No	2781	84.9	24.5	806	85.5	24.4
Yes	493	15.1	28.2	137	14.5	28.5
Smoking						
No	2852	87.1	23.6	847	89.8	23.8
Yes	422	12.9	34.8	96	10.2	35.4
Binge drinking						
No	2604	79.5	22.5	528	56.0	22.9
Yes	670	20.5	34.9	415	44.0	27.7
Insomnia symptoms						
No	2711	82.8	20.9	811	86.0	21.2
Yes	563	17.2	44.9	132	14.0	48.5

* Poor mental health functioning was defined as belonging to the lowest quartile (Q1) of the mental component summary (MCS).

Mental health functioning was the lowest among those with adverse psychosocial working conditions. Participants reporting exposure to high job demands and low job control reported lower mental health functioning than their less-exposed counterparts. In addition, mental health functioning was slightly lower among women who reported adverse physical working conditions.

### Main results

We found clear associations between psychosocial working conditions and mental health functioning (Table II). In the first model for job demands, those with high job demands had 1.79 (95% CI = 1.55-2.07) times higher odds for poor mental health functioning than those with low job demands after adjusting for sociodemographic characteristics. The adjustments for the other exposures, poor health and adverse health behaviours in Models 2 and 3 attenuated the association only slightly. After all adjustments, those with high job demands were, on average, still more likely (OR = 1.69; 95% CI = 1.45-1.97) to have poor health functioning compared with those with low job demands.

**Table II.** The associations between job control, job demands and physical workload with poor mental health functioning, odds ratios (OR) with their 95% confidence intervals (95 % CI).

	Model 1	Model 2	Model 3	Model 4
	OR* (95 % CI)	OR (95 % CI)	OR (95 % CI)	OR (95 % CI)
Working conditions				
Job demands				
*Low*	1.00	1.00	1.00	1.00
*High*	1.78 (1.54-2.06)	1.78 (1.54-2.06)	1.66 (1.43-1.92)	1.69 (1.45-1.97)
Job control				
*High*	1.00	1.00	1.00	1.00
*Low*	1.69 (1.44-1.97)	1.67 (1.43-1.96)	1.63 (1.39-1.92)	1.65 (1.40-1.94)
Physical workload				
*Low*	1.00	1.00	1.00	1.00
*High*	1.17 (0.98-1.40)	0.96 (0.80-1.16)	1.04 (0.87-1.25)	0.87 (0.72-1.05)

* OR = Odds ratio for poor mental health functioning (lowest quartile of mental component summary) at given level for working conditions.

Model 1: Working conditions individually adjusted for gender, age, marital status, having children, and occupational class.

Model 2: Model 1 adjusted for all working conditions simultaneously.

Model 3: Model 1 adjusted for poor health (obesity and insomnia) and adverse health behaviours (alcohol consumption and smoking).

Model 4: Adjusted for all covariates.

Job control was also associated with mental health functioning. In Model 1, those with low job control were more likely (OR = 1.69; 95% CI = 1.44-1.97) to have poor mental health functioning than their counterparts with high job control when sociodemographic characteristics were adjusted for. Adjustments for the other exposures, poor health and adverse health behaviours did not result in substantial changes in the estimates. In the full model, those with low job control had 1.64 (95% CI = 1.40-1.94) times higher odds for poor mental health functioning than those with high job control.

Having a high physical workload indicated a slightly higher likelihood of having poor mental health functioning compared with those with low physical workload in the first model (OR = 1.17; 95% CI = 0.98-1.40). Throughout the Models 2–4, the estimate turned statistically non-significant, producing the fully adjusted estimate of 0.87 (95% CI = 0.72-1.05).

All sensitivity analyses produced estimates that were qualitatively similar to those presented. When reproducing the estimates using data with missing cases or without including occupational class in the models, the results changed only marginally with just one exception where physical workload became statistically significant in Model 1 in the analysis using missing cases. Analyses using four-level independent variables did not change the inferences from the main analyses.

## Discussion

We studied the associations between adverse working conditions and mental health functioning among municipal employees below the age of 40. The results showed that adverse psychosocial working conditions were associated with poor mental health functioning after adjusting for sociodemographic characteristics, poor health, adverse health behaviours and physical working conditions. After the full adjustments, we found odds for poor mental health functioning to be 1.7 times higher for those with high job demands compared with those with low job demands. In a similar way, the corresponding odds were 1.7 for those with low job control when compared to those with high job control. After the adjustments, adverse physical working conditions were not associated with lower mental health functioning.

Our results align with the earlier studies that have observed associations of high job demands and low job control with mental disorders [5–7]. Some previous studies have found the association only for low job control [29], e.g. that job control would have a buffering effect against the adverse effects high job demands might have on the employee [30]. In this study, we found the association for both job control and job demands but found no interaction effects between the variables. We extend the current knowledge base by focusing on younger employees and by using mental health functioning as the outcome.

Despite the earlier evidence that physical working conditions may be associated with mental disorders [10, 11], in this study, we did not find an association. The result may reflect that persons with adverse physical working conditions also experience adverse psychosocial working conditions. However, in contrast to recent studies exploring joint contributions of physical and psychosocial working conditions on mental disorders [31, 32], we did not find any interaction effect between these variable categories. However, we did observe modest correlations between continuous physical workload and job control (r=0.25) and physical workload and job demands (r=-0.29) (Appendix 3), but it was beyond the scope of this study to explore these associations further. We wish to interpret these results with caution. It is also reasonable to assume that the association between adverse physical working conditions and mental health functioning accumulates over time. In our cohort of younger employees, the participants may not have been exposed to adverse physical working conditions for a long enough time for this association to emerge. These types of results might be better captured in longitudinal studies, which would provide more robust inferences.

We also wish to note that the knowledge on different possible underlying mechanisms behind the observed associations is inconclusive. Some studies indicate that the effects of working conditions on common mental disorders may differ between women and men [5]. Other studies, including a recent meta-analysis, showed no gendered pattern in the association between working conditions and depressive symptoms [7]. In this study, the average score for MCS and threshold for poor mental health functioning were 1 to 1.5 points lower in women than in men. Despite this, we did not find gender-stratified effects which is in line with an earlier meta-analysis. Gender did not modify the associations between adverse working conditions and the associations were similar for women and men [7]. However, this result needs to be interpreted cautiously, as in the present study a high number of the participants work in female-dominated human service occupations in which there is an increased risk for sickness absence due to mental disorders [20, 33]. Furthermore, we cannot rule out gender related non-response bias as response rate to the questionnaire was lower for men (48%) than for women (53%), and in this study particularly the age distribution of the group of men below 30 years of age deviates from the distribution of the target population [14].

Our results showed that poor health and adverse health behaviours may explain a proportion of the association between adverse psychosocial working conditions and mental health functioning. Judging the direction of causality is inconclusive; however, individuals with adverse working conditions may be more prone to adopt poorer health behaviours [34]. The question of reverse causation is present in addition to other confounding factors. Nevertheless, the meta-analyses suggest that the associations between working conditions and mental health outcomes are likely to hold in high-quality longitudinal study settings [5–7].

This study is one of the first studies conducted using the first wave of the new Young Helsinki Health Study cohort. The study uses well-established measures [15], and the participants are representative of the study population [14]. Nevertheless, the study has a number of limitations. First, the study design was cross-sectional. Although meta-analyses [5–7] have shown a robust association between adverse working conditions and poor mental health, caution on causal inference is warranted, especially when drawing inferences from a cross-sectional data [35]. There is a possibility of reverse causation as lower mental health functioning might influence the reporting of occupational factors and lead to an adverse experience of working conditions [10]. Second, as a survey, this study may suffer from biases related to non-response [14], and non-response to items on the questionnaire. Third, our measures on poor health and adverse health behaviours were based on self-reports and, therefore, potentially underreported. Fourth, the SF-36 is a self-administered instrument and not a clinical diagnostic interview administered. The risk of reporting bias increases when both exposures and outcome are self-reported [36]. Fifth, using dichotomous variables may lead to invalid inferences [37]. We have aimed to reduce this risk by using cut-off points that have been commonly used in previous studies [7, 10, 27], by being explicit about the choices involved and by inspecting that the inferences hold in the sensitivity analyses (please see Appendices 1–2). Sixth, we studied only a selection of work-related characteristics. Other known psychosocial aspects, namely organisational injustice, high effort-reward imbalance, role stress, organisational change, low social support, workplace conflict and bullying and job characteristics, such as job insecurity, atypical working hours and employment status [5–7], were not studied, and it is important to include these factors in future studies on mental health functioning and work ability among younger employees. Finally, our data consisted of public sector employees and no comparison with other employment sectors could be conducted. This may be considered a limitation to the generalizability of the results as public sector work may contain characteristics that affect both the experiences of working conditions and mental health functioning [20, 33]. Despite these limitations, our results justify giving special attention to psychosocial working conditions and mental health functioning of younger employees who should still have long careers and many productive years ahead.

## Conclusions

Psychosocial working conditions were associated with mental health functioning in younger employees. Impaired functioning is likely to cause health-related lost productivity, and may lead to work disability. Future studies focusing on mental health functioning are warranted as they can advance the understanding of the complex interplay between working conditions, mental health functioning and work disability.

## Supplemental Material

sj-docx-1-sjp-10.1177_14034948211045458 – Supplemental material for Working conditions and mental health functioning among young public sector employees Click here for additional data file. Supplemental material, sj-docx-1-sjp-10.1177_14034948211045458 for Working conditions and mental health functioning among young public sector employees by Jaakko Harkko, Hertta Ranta, Tea Lallukka, Hilla Nordquist, Minna Mänty and Anne Kouvonen in Scandinavian Journal of Public Health
