# Supplementary material for: Working conditions and mental health functioning among young public sector employees
Source: Scand J Public Health. 2021 Oct 5;51(1):98–105. doi: 10.1177/14034948211045458 (PMC9900189; doi:10.1177/14034948211045458)
Supplement: sj-docx-1-sjp-10.1177_14034948211045458 – Supplemental material for Working conditions and mental health functioning among young public sector employees [file sj-docx-1-sjp-10.1177_14034948211045458.docx]

Appendix 1. The associations between job control, job demands and physical workload with poor mental health functioning. Results from full sample with missing data (n=5898)

|  | Model 1 | Model 2 | Model 3 | Model 4 |
| --- | --- | --- | --- | --- |
|  | OR (95 % CI) | OR (95 % CI) | OR (95 % CI) | OR (95 % CI) |
| Working conditions |  |  |  |  |
| Job demands |  |  |  |  |
| *Low* | 1.00 | 1.00 | 1.00 | 1.00 |
| *High* | 1.83 (1.61-2.09) | 1.83 (1.59-2.09) | 1.65 (1.44-1.90) | 1.67 (1.45-1.93) |
| Job control |  |  |  |  |
| *High* | 1.00 | 1.00 | 1.00 | 1.00 |
| *Low* | 1.55 (1.34-1.78) | 1.53 (1.33-1.77) | 1.55 (1.33-1.80) | 1.55 (1.34-1.80) |
| Physical workload |  |  |  |  |
| *Low* | 1.00 | 1.00 | 1.00 | 1.00 |
| *High* | 1.23 (1.05-1.44) | 1.00 (0.85-1.18) | 1.13 (0.95-1.33) | 0.93 (0.79-1.11) |

Appendix 2 The associations between job control, job demands and physical workload with poor mental health functioning, odds ratios (OR) with 95% confidence intervals (95 % CI). Analyses using four-class independent variables.

|  |  | Model 1 | Model 2 | Model 3 | Model 4 |
| --- | --- | --- | --- | --- | --- |
|  | N (%) | OR* (95 % CI) | OR (95 % CI) | OR (95 % CI) | OR (95 % CI) |
| Working conditions |  |  |  |  |  |
| Job demands |  |  |  |  |  |
| *1st Low* | 1085 (25.7) | 1.00 | 1.00 | 1.00 | 1.00 |
| *2nd* | 1220 (28.9) | 0.95 (0.77-1.17) | 0.97 (0.78-1.19) | 0.91 (0.74-1.13) | 0.94 (0.76-1.16) |
| *3rd* | 874 (20.7) | 1.26 (1.02-1.57) | 1.29 (1.03-1.61) | 1.20 (0.96-1.49) | 1.24 (0.99-1.56) |
| *4th High* | 1038 (24.6) | 2.21 (1.81-2.70) | 2.22 (1.80-2.73) | 1.96 (1.60-2.40) | 2.02 (1.63-2.50) |
| Job control |  |  |  |  |  |
| *1st High* | 1021 (24.2) | 1.00 | 1.00 | 1.00 | 1.00 |
| *2nd* | 999 (23.7) | 1.26 (1.01-1.57) | 1.28 (1.02-1.60) | 1.24 (0.99-1.56) | 1.27 (1.01-1.60) |
| *3rd* | 1077 (25.5) | 1.65 (1.32-2.06) | 1.68 (1.34-2.10) | 1.60 (1.27-2.01) | 1.65 (1.31-2.07) |
| *4th Low* | 1120 (26.6) | 2.25 (1.80-2.81) | 2.20 (1.75-2.76) | 2.14 (1.70-2.69) | 2.14 (1.70-2.71) |
| Physical workload |  |  |  |  |  |
| *1st Low* | 1338 (31.7) | 1.00 | 1.00 | 1.00 | 1.00 |
| *2nd* | 843 (20.0) | 1.06 (0.86-1.30) | 0.99 (0.80-1.22) | 1.08 (0.88-1.34) | 1.02 (0.82-1.27) |
| *3rd* | 1065 (25.3) | 1.15 (0.94-1.41) | 0.98 (0.79-1.20) | 1.12 (0.91-1.38) | 0.97 (0.78-1.20) |
| *4th High* | 971 (23.0) | 1.27 (1.02-1.57) | 0.92 (0.73-1.16) | 1.12 (0.90-1.40) | 0.84 (0.66-1.07) |

* OR = Odds ratio for poor mental health functioning (lowest quartile of Mental Component Summary) at given level for working conditions

Model 1: Working conditions individually adjusted for gender, age, marital status, having children, and occupational class

Model 2: Model 1 adjusted for all working conditions simultaneously

Model 3: Model 1 adjusted for poor health (obesity and insomnia) and adverse health behaviours (alcohol consumption and smoking)

Model 4: Adjusted for all covariates

Appendix 3. Pearson's Correlation matrix for the study variables (as continuous variables)

|  | 1. | 2. | 3. | 4. |
| --- | --- | --- | --- | --- |
| 1. Job demands | 1.0000 |  |  |  |
| 2. Job control | 0.0085 | 1.0000 |  |  |
| 3. Physical workload | 0.2526 | -0.2870 | 1.0000 |  |
| 4. Mental health functioning | -0.0948 | 0.0919 | -0.0166 | 1.0000 |
